# Outcomes of changing systemic therapy in patients with relapsed breast cancer and 1 to 3 brain metastases

**DOI:** 10.1038/s41523-021-00235-7

**Published:** 2021-03-19

**Authors:** Omar Alhalabi, Zaid Soomro, Ryan Sun, Elshad Hasanov, Aya Albittar, Debu Tripathy, Vicente Valero, Nuhad K. Ibrahim

**Affiliations:** 1 Division of Cancer Medicine, The University of Texas MD Anderson Cancer Center, Houston, TX USA; 2 Departments of Neuro-Oncology, The University of Texas MD Anderson Cancer Center, Houston, TX USA; 3 Biostatistics, The University of Texas MD Anderson Cancer Center, Houston, TX USA; 4 Investigational Therapeutics, The University of Texas MD Anderson Cancer Center, Houston, TX USA; 5 Breast Medical Oncology, The University of Texas MD Anderson Cancer Center, Houston, TX USA; 6 Present Address: Department of Medicine, the State University of New York Upstate Medical University, Syracuse, NY USA

**Keywords:** Breast cancer, Targeted therapies, Metastasis

## Abstract

The development of brain metastases (BMs) in breast cancer (BC) patients remains a challenging complication. Current clinical practice guidelines recommend local treatment of BMs without changing systemic therapy (CST) in patients with stable extracranial disease. We retrospectively investigated the impact of CST (when applicable as per treating physician’s discretion) following the diagnosis and management of oligometastatic (1–3) BMs in patients without extracranial metastases on the progression-free survival time (PFS), and overall survival (OS). Hazard ratios (HRs) were calculated using the Cox proportional hazard model. Among the 2645 patients with BC and BMs treated between 2002 and 2015, 74 were included for analysis. 40.5% of patients had HER2 + disease. Median time from diagnosis of BC to BMs was 17.6 months. 54%, 8%, and 38% of BMs were managed by radiation, craniotomy, or combination, respectively. Following the primary management of BMs, we observed that CST occurred in 26 (35.5%) patients, consisting of initiation of therapy in 13.5% and switching of ongoing adjuvant therapy in 22%. Median PFS was 6.6 months among patients who had CST compared to 7.1 months in those who did not (HR = 0.88 [0.52–1.47], *p* = 0.62). Median OS was 20.1 months among patients who had CST compared to 15.1 months in those who did not (HR = 0.68 [0.40–1.16], *p* = 0.16). Upon the successful local management of oligometastatic BMs in patients without extracranial disease, we did not find a significant difference in survival between patients who experienced a change in systemic therapy as compared to those who did not.

Despite advances in systemic therapies and improved overall survival of metastatic breast cancer (MBC) patients, the development of brain metastases (BMs) remains a challenging complication in 10–20% of patients and affects the quality of life and increases morbidity and mortality^1,2^. About 3% of localized breast cancer (BC) patients are reported to relapse with BMs without extracranial disease^3^. Radiation therapy, with either whole brain radiation therapy (WBRT), or stereotactic radiosurgery (SRS), with or without surgical resection, are the current gold-standard first-line treatment for patients with BMs without extracranial disease^4^. In patients with 1–3 BMs, two phase III trials showed that adjuvant SRS is an effective modality after surgical resection that spares the patients the toxic effect of WBRT^5,6^. Furthermore, for patients who have 1–3 BMs with no evidence of extracranial disease and achieve an excellent clinical response after treatment for BMs, there are no retrospective or prospective data to demonstrate the role of systemic treatment. In fact, the current clinical practice guidelines, in patients with Human Epidermal Growth Factor Receptor 2–Positive (HER2+) disease, recommend local treatment of BMs without changing concurrent systemic therapy (CST) in patients with stable extracranial disease^7^. Here, we retrospectively investigated the impact of CST (when applicable as per treating physician’s discretion) after successful local management of the 1-3 BMs on the patient’s progression-free survival time (PFS) and on the patient’s overall survival (OS).

We included 74 of 2645 (3%) patients who had complete electronic medical records to meet our inclusion criteria were identified in our institutional database. Following the primary management of BM, we observed that treating medical oncologists changed systemic therapy in 26 of 74 (35.5%), defined as the CST group (Table 1). CST comprises both initiation of therapy in 10 of 74 (13.5%) and switching of adjuvant therapy in 16 of 74 (22%) (Fig. 1). At baseline, 44 of 74 (60%) patients were not receiving any systemic therapy (Table 2). After the local management of BMs, 34 of 44 (78%) stayed off systemic therapy, while 10 of 44 (22%) were initiated on systemic therapy. Table 3 summarizes the CST strategies (*n* = 26), which included the initiation of lapatinib in 7 patients (27%).

**Table 1:** Baseline characteristics of analyzed patients.

Parameter	Total (*n* = 74)	CST (*n* = 26)	No-CST (*n* = 48)	*P Value*
Median age at diagnosis of brain metastasis (years)	52	53.7	49.9	0.389
Time from diagnosis of breast cancer to diagnosis of brain metastasis (months)	17.6	20.2	17.41	0.150
Year of brain metastasis diagnosis, median (range)	2008 (2002–2015)	2008 (2002–2015)	2009 (2002–2014)	na
Histological type (primary breast cancer) (%)				0.229
Ductal	68 (92)	23 (88)	45 (94)	
Lobular	2 (3)	2 (8)	0 (0)	
Others	4 (5)	1 (4)	3 (6)	
Grade (primary breast cancer) (%)				0.04
II	13 (18)	8 (31)	5 (10.4)	
III	58 (78)	18 (69)	40 (83.3)	
Missing	3 (4)	0 (0)	3 (6.2)	
Tumor subtype (primary breast cancer) (%)				0.006
HR+/HER2−	17 (23)	12 (46.2)	9 (19.6)	
HR+/HER2+	12 (16)	6 (23.1)	6 (13.0)	
HR−/HER2+	18 (24)	6 (23.1)	12 (26.1)	
HR−/HER2−	21 (28)	2 (7.7)	19 (41.3)	
Missing	2 (3)	0 (0)	2 (4)	
Number of brain metastasis				0.627
Solitary (%)	42 (57)	16 (62)	26 (54)	
Two or three (%)	32 (43)	10 (38)	22 (46)

**Fig. 1:**
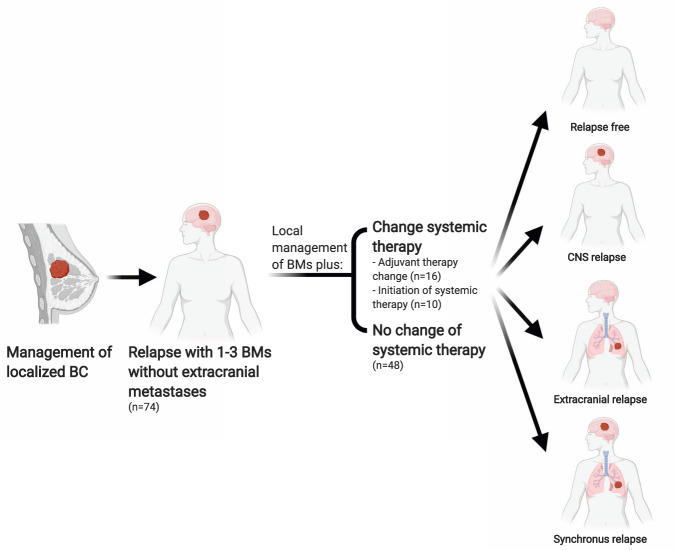
Clinical scenarios of analyzed patients. BC, breast cancer. BM, brain metastases. CNS, central nervous system.

**Table 2:** Systemic management for analyzed patients.

*Baseline systemic therapy (%)*	*n* = 74
Adjuvant endocrine	19 (26)
Adjuvant chemotherapy	2 (3)
Adjuvant trastuzumab	8 (11)
Adjuvant investigational therapy	1 (1)
None	44 (59)
*Systemic therapy after local management (%)*	*n* = 74
Endocrine	22 (30)
Chemotherapy	5 (7)
HER2-directed therapy	5 (7)
Endocrine/HER2-directed therapy	3 (4)
Chemotherapy/HER2-directed therapy	4 (5)
None	35 (47)

**Table 3:** Change of systemic therapy strategies.

CST strategies	Total = 26
*Starting or switching endocrine therapy*	14 (54)
None → AI	3 (11.5)
None → Tamoxifen	2 (8)
Tamoxifen → AI	2 (8)
Tamoxifen → Capecitabine + lapatinib	1 (4)
AI → Another AI	4 (15)
AI → Capecitabine	1 (4)
AI → AI + lapatinib	1 (4)
*Starting or switching chemotherapy*	3 (11.5)
None → Capecitabine	2 (8)
Taxane → AI	1 (4)
*Starting or switching HER2 therapy*	9 (35)
None → Lapatinib ± capecitabine	3 (11.5)
Trastuzumab → Trastuzumab + tamoxifen	1 (4)
Trastuzumab → Trastuzumab + AI	1 (4)
Trastuzumab → Trastuzumab + lapatinib	1 (4)
Trastuzumab → Capecitabine + lapatinib	1 (4)
Trastuzumab → None	1 (4)
Lapatinib → Capecitabine	1 (4)

Baseline characteristics are summarized in Table 1. The median age at the time of BM diagnosis was 52.0 years. The median time from diagnosis of BC to developing BMs was 17.6 months. 40.5% of patients were with HER2 + disease. 57% of patients had a solitary BM. Baseline characteristics were not significantly different among the CST and no-CST groups except for hormonal status and histological grade. Hormone receptor positive patients were more common among the CST group (46.2%) compared the no-CST group (19.6%). On the other hand, patients with triple negative BC were more common among the no-CST group (41.3%) compared to CST group (7.7%) (*p* = 0.006). Histological grade was higher among the no-CST group (*p* = 0.04).

In regard to primary BMs management in patients (Supplementary Table 1), 40 of 74 (54%) were treated by definitive radiation therapy, which included 18 patients treated with SRS, 11 with WBRT, and 11 with SRS plus WBRT. On the other hand, 34 of 74 (46%) patients were managed by surgical resection and in 28 of 34 (82%), this was followed by adjuvant radiation therapy. Adjuvant SRS was used in 6 of 28 (21%) patients, while adjuvant WBRT was used in 22 of 28 (79%) patients. There was a trend toward utilizing adjuvant SRS more than WBRT in recent compared to prior years. The median year of using SRS as adjuvant therapy was 2013 (range: 2008-2016), while median year of WBRT as adjuvant therapy was 2009 (range: 2002–2014). All patients had staging imaging documenting lack of extracranial metastases at the time of local therapy of BMs.

Median OS for all patients was 16.8 months (95% CI: 13.7–27.1). OS among CST was 20.1 months vs. 15.1 months among no CST (HR = 0.68, 95% CI: [0.40–1.17], *p* = 0.16) (Fig. 2a). Median PFS for all patients was 7 months (95% CI: 6.3–10.7). Median PFS among patients who had CST was 6.6 months as compared to 7.1 among those who had no-CST (HR = 0.88 [0.52–1.47], *p* = 0.62) (Fig. 2b). Extracranial PFS among patients who had CST was 14.9 months (95% CI 10–30.5) as compared to 11.6 months among with no-CST patients (95% CI 8.3–19.8) (hazard ratio 0.81, 95% CI 0.48–1.37, *p*-value 0.44) (Fig. 2c). Patterns of intracranial and extracranial relapse are summarized in Supplementary Table 2. 5-year RMST for the CST group was 15.9 months vs. 12.5 months in no CST group. The difference of 3.4 months (95% CI −5.2 to 12.1) was not statistically significant (*p* = 0.36). Among all patients (*n* = 74), median OS in HR + /HER2− was 27.1 months [14.8–45.5], in HR+/HER2+ 48.1 months [19.1-NA], in HR-/HER2+ 12.9 months [7.6–38.3] and in HR−/HER2− 16.7 months [8.6–35.7]. OS in solitary BM was 24.3 months [16.8–42.3] and in 2–3 BM was 11.9 months [7.5–17.8].

**Fig. 2:**
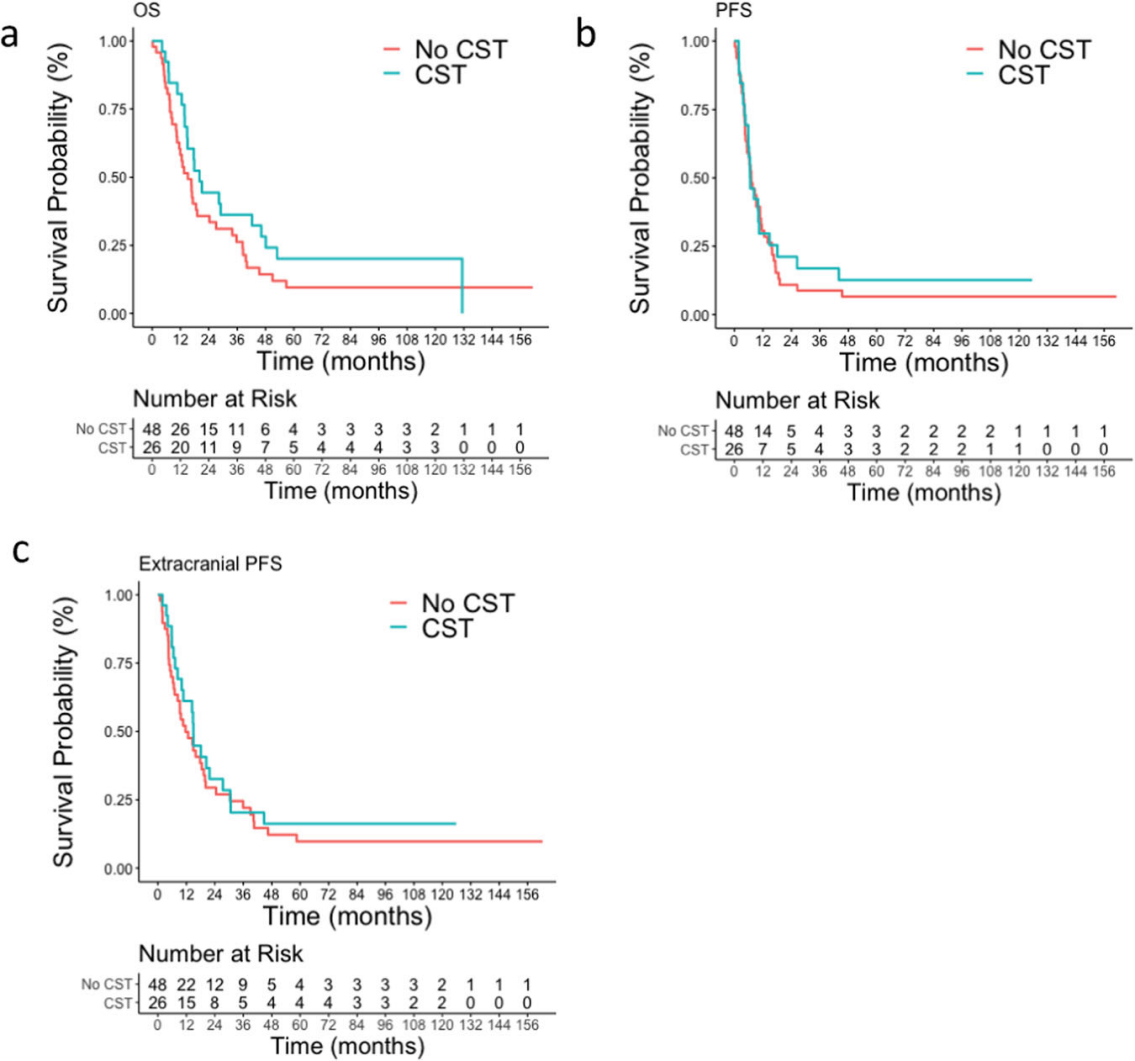
Survival comparison between patients who had change of systemic therapy (CST) and those who did not (no-CST). **a** Median OS was 20.1 months among patients who had CST as compared to 15.1 among those who had no-CST (HR = 0.68 [0.40–1.16], *p* = 0.16). **b** Median PFS was 6.6 months among patients who had CST as compared to 7.1 among those who had no-CST (HR = 0.88 [0.52–1.47], *p* = 0.62). **c** Extracranial PFS from local management of BM in CST patients (14.9 months; 95% CI 10–30.5) compared with no-CST patients (11.6 months; 95% CI 8.3–19.8) (hazard ratio 0.81, 95% CI 0.48–1.37, *p*-value 0.44).

The effects of systemic chemotherapy after the management of isolated metastatic brain lesions are poorly defined and only anecdotally reported. In the context of co-existing extracranial disease, Lee et al reported that the median OS of BC patients with BMs improved from 3.6 months to 7.8 months with systemic chemotherapy^8^. This OS benefit was also found in other studies, which highlighted the fact that improved OS might be attributable to efficient control of intracranial and extracranial disease^9^. However, the impact of initiating systemic therapy or changing adjuvant therapy in the context of isolated 1–3 BMs has not yet been defined.

In our analysis, 74 patients with BC relapse as 1–3 BMs without the extracranial disease had a median OS of 16.8 months, and median PFS of 7 months after local therapy, which seems longer than prospective trials showing median OS of 12.2 months and a median time to intracranial tumor progression of 6.4^6^. This somewhat longer OS in our cohort could be explained by the fact that these patients had no systemic disease and only oligometastatic CNS disease. Findings from our single institution, long-term follow-up, retrospective cohort study evaluate the potential role of initiating systemic therapy or changing adjuvant therapy on PFS and OS in patients with BC and 1–3 BMs. Median OS and extracranial PFS were longer, but non-statistically significant, for CST compared to no-CST group (20.1 vs. 15.1 months), and extracranial PFS (14.9 vs. 11.6), respectively. 5-year RMST for the CST group was also longer but non-statistically significant (15.9 vs. 12.5 months) for CST versus no-CST group.

We observed that among 537 patients with BC and 1–3 BMs, 74 (14%) patients had BMs with no extracranial metastases, which is approximate to an analysis of 1712 patients with BC metastatic to the brain, where BMs were the first manifestation of metastatic disease without extracranial disease in 20% of patients^10^. Furthermore, we observed that the median time from diagnosis of BC to diagnosis of BMs was 17.6 months, which is close to a previously published report of a median of 12.8 months after diagnosis of BC^11^. Of note, the subtype distribution in our study showed 40% HER2+ and 28% triple negative (TN), which is compatible with a study published previously by our group from a larger cohort of 873 patients with BMs showing 39.4% HER2+ and 34.1% TN subtypes (Gao et al. Int. J. Cancer. 2020—in print).

Our study has a few limitations that we highlight here to help the reader better put the findings into context. First, because this is a single-center retrospective study with no randomization, the results are not conclusive; rather, they are hypothesis generating. In our study there were significant imbalances between the CST and no-CST group regarding tumor subtype; however, due to the small sample sizes, it was not possible to adjust for possible confounding factors in multivariate analysis, which could be an area for future analysis. Second, a limited sample size of patients that have 1–3 BMs and no extracranial disease at the initial presentation. Third, the study spanned different systemic chemotherapy settings, introducing further bias. In 7 of 26 (27%) patients in the CST group, lapatinib was initiated to control CNS disease. Lapatinib plus capecitabine or lapatinib alone have been quoted an overall CNS response rate (RR) between 21% and 29%^12^. Nonetheless, newer antiHER2 agents not included in our timeframe (2002–2015) such as trastuzumab-emtansine (T-DM1) (CNS RR 44%), neratinib (CNS RR 49%), or tucatinib (Intracranial RR 47%) show higher intracranial activity^9,13–15^, which limits the generalization of our results in the current treatment landscape for HER2+ disease. Well-designed randomized controlled trials using these newer CNS-active agents are urgently needed to assess systemic treatment modalities for BC patients with oligometastatic brain involvement and stable extracranial disease.

Our findings did not demonstrate a statistically significant difference in OS or PFS between patients who experienced a change in systemic therapy compared to those who did not. However, in light of contemporary agents that have notable activity against CNS disease, more prospective efforts are needed to investigate the best systemic therapy approach upon developing isolated BMs.

## Methods

### Patients

We identified 2645 consecutive patients with biopsy-proven BC and initial image-proven BMs who were diagnosed at our institution between January 1, 2002 and December 30, 2015. We included patients with previously treated localized BC who are relapsing with 1–3 BMs without the extracranial disease (Fig. 1).

Patient information was collected from the electronic medical record database, including demographics, histological type, hormone receptor (HR) and human epidermal growth factor receptor 2 (HER2) status, number of BMs, lack of extracranial disease at the time of BMs diagnosis, date of BMs diagnosis and subsequently confirmed disease progression in the brain or extracranially, the local therapy patients received after BMs diagnosis, the systemic therapy changes after BMs diagnosis and clinical outcomes. Patients with >3 BMs, or concurrent extracranial disease (*n* = 446), and duplicate or missing treatment data (*n* = 17) were excluded (Supplemental Fig. 1).

At the discretion of the treating physician, patients underwent WBRT, SRS, SRS followed by WBRT (SRS + WBRT), surgical resection, or surgical resection followed by radiation (SRS or WBRT) as local therapy. We investigated the impact of CST (when applicable as per treating physician’s discretion) after diagnosis of the initial 1-3 BMs on the patient’s progression-PFS, defined as time to death, development of a second BMs or an extracranial spread. Where indicated and as per treating physician discretion, patients were defined as the CST group if they had their adjuvant chemotherapy switched after the occurrence of BM, or had systemic therapy initiated aiming at maintaining the lack of development of the extracranial metastatic disease, following the documentation of BM. Patients were followed up and underwent imaging every 2–3 months until death or disease progression in BMs or extracranially; data were censored as of December 30, 2019.

Our investigation was approved by the institutional review board (IRB) of our institution. Waiver of informed consent was granted by the IRB due to the retrospective nature of our study.

### Statistical analysis

Overall survival (OS) as calculated from the time from BM diagnosis to death from any cause. OS was censored for patients who were alive at last contact. Progression free survival (PFS) was defined as the time from definitive local management of the BM to another intracranial (progression of locally treated metastases in the brain, distant disease at a new site in the brain, or leptomeningeal disease), extracranial relapse or death. PFS was censored in patients who were alive and without progression at last follow up. Extracranial PFS was defined as the time from definitive local management of the BM to extracranial relapse, or death. Extracranial PFS was censored for patients without extracranial relapse at last follow-up. OS, PFS, and extracranial PFS were estimated by using the Kaplan–Meier method and compared across group using the log-rank test. We also computed the restricted mean survival time (RMST) up to 5 years of follow-up. Univariate Cox proportional hazards analyses were performed to evaluate the effects of CST on OS, PFS, and extracranial PFS. The hazard ratio (HR) was estimated with 95% confidence interval (CI). R version 3.6.1 software was used for the analysis.

### Reporting summary

Further information on research design is available in the Nature Research Reporting Summary linked to this article.

## Supplementary information

Supplementary Information

Reporting Summary Checklist

## Data Availability

The data that support the findings of this study are not publicly available, but will be made available on reasonable request from the corresponding author, Dr. Nuhad K Ibrahim, email address: nibrahim@mdanderson.org. The data generated and analysed during this study are described in the following metadata record: 10.6084/m9.figshare.13713202^16^.

## Code availability

No custom codes were used in analysis reported in this study. All relevant references are provided in the methods section.
