# Supplementary material for: Outcomes of changing systemic therapy in patients with relapsed breast cancer and 1 to 3 brain metastases
Source: NPJ Breast Cancer. 2021 Mar 19;7:28. doi: 10.1038/s41523-021-00235-7 (PMC7979865; doi:10.1038/s41523-021-00235-7)

Supplementary Table 1: Local therapy approaches for analyzed patients.

| **Parameter (%)** | **Total (n=74)** | **CST (n=26)** | **No CST (n=48)** | ***P Value*** |
|---|---|---|---|---|
| Local management | | | | 1 |
| Definitive radiation | 40 (54) | 14 (54) | 26 (54.2) | |
| Surgery | 6 (8) | 2 (8) | 4 (8.3) | |
| Surgery followed by adjuvant radiation | 28 (38) | 10 (38) | 18 (37.5) | |
| Definitive radiation strategy | | | | 0.40 |
| SRS | 18 (24) | 8 (31) | 10 (21) | |
| SRS + WBRT | 11 (15) | 4 (15) | 7 (14.6) | |
| WBRT | 11 (15) | 2 (15.6) | 9 (18.75) | |
| Adjuvant radiation strategy | | | | 1 |
| SRS | 6 (7.5) | 2 (6.25) | 4 (8.3) | |
| WBRT | 22 (27.5) | 8 (25) | 14 (29.2) | |

Supplementary Table 2: Patterns of first relapse after successful management of BMs

| Total (%) | **All subtypes* N=57** | **HR+ HER2- N=18** | **HR+ HER2+ N=8** | **HR- HER2+ N=14** | **HR- HER2- N=15** |
|---|---|---|---|---|---|
| **CNS (%)** | 36 (67) | 10 (55) | 6 (75) | 9 (64) | 9 (60) |
| Brain[Δ] | 28 | 8 | 4 | 6 | 8 |
| Leptomeningeal disease | 8 | 2 | 2 | 3 | 1 |
| **Extracranial (%)** | 12 (19) | 3 (16) | 2 (25) | 5 (36) | 2 (13) |
| Lymphadenopathy | 5 | 3 | 0 | 1 | 1 |
| Lung | 3 | 0 | 2 | 1 | 0 |
| Bone | 2 | 0 | 0 | 2 | 0 |
| Liver | 1 | 0 | 0 | 0 | 1 |
| Other | 1 | 0 | 0 | 1 | 0 |
| **Synchronous: CNS + extracranial (%)** | 9 (14) | 5 (28) | 0 | 0 | 4 (27) |

* two patients had unknown HER2 status; therefore, they are not representing the subtypes columns.

[Δ] among the 28 patients who had a relapse in the brain, 15 patients relapsed in the form of new lesions and 13 relapsed at the previously treated site.

Supplemental Figure 1: Consort diagram of the inclusion and exclusion criteria of analysis.

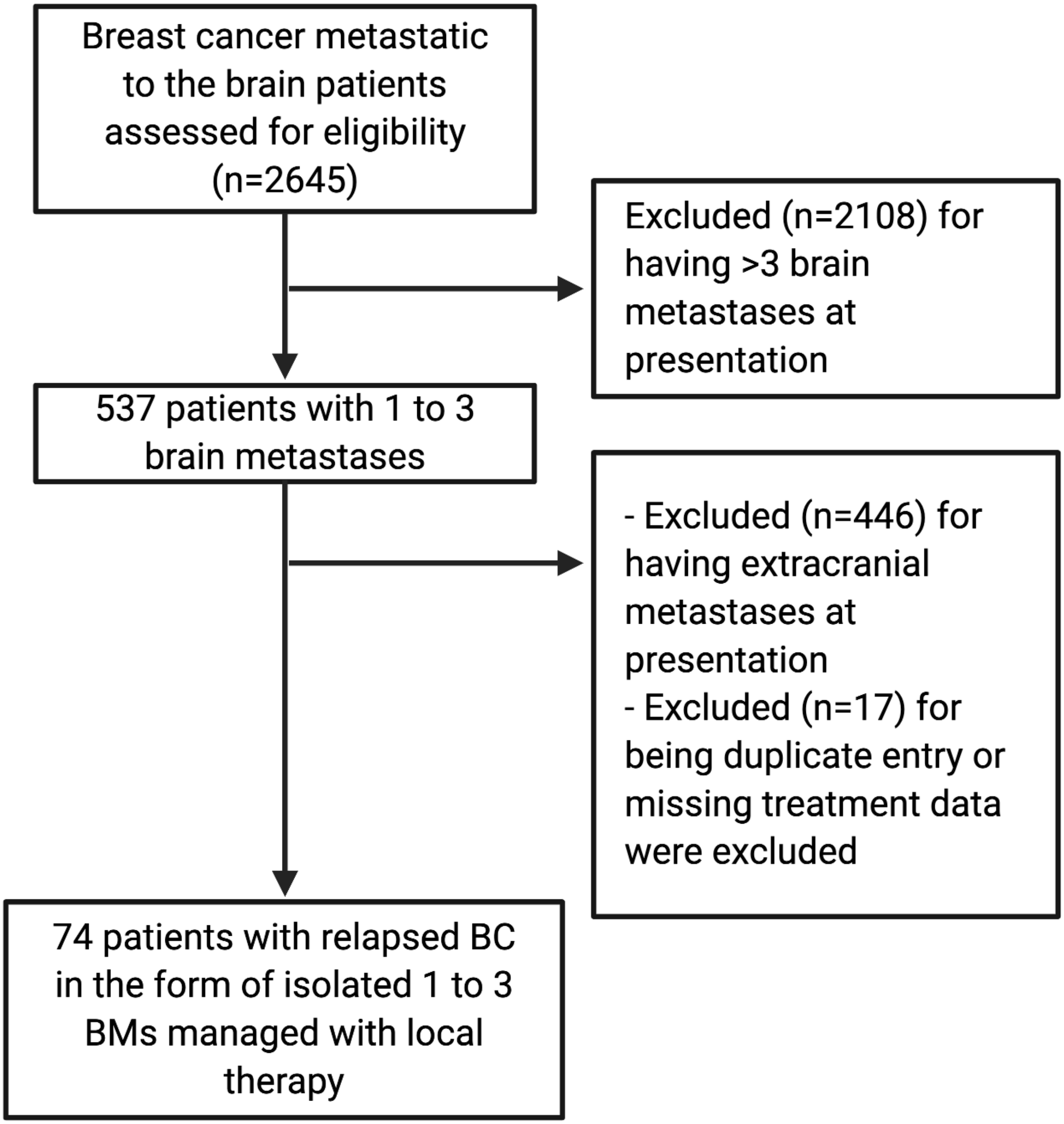

Supplement: Supplementary file 1 — Supplementary Information [file 41523_2021_235_MOESM1_ESM.pdf]
