# Supplementary material for: Outcomes of changing systemic therapy in patients with relapsed breast cancer and 1 to 3 brain metastases
Source: NPJ Breast Cancer. 2021 Mar 19;7:28. doi: 10.1038/s41523-021-00235-7 (PMC7979865; doi:10.1038/s41523-021-00235-7)
Supplement: Supplementary file 2 — Reporting Summary Checklist [file 41523_2021_235_MOESM2_ESM.pdf]

nature research

Corresponding author(s): Nuhad Ibrahim

Last updated by author(s): Jan 24, 2021

Reporting Summary

Nature Research wishes to improve the reproducibility of the work that we publish. This form provides structure for consistency and transparency in reporting. For further information on Nature Research policies, see our Editorial Policies and the Editorial Policy Checklist.

Statistics

For all statistical analyses, confirm that the following items are present in the figure legend, table legend, main text, or Methods section.

n/a	Confirmed	
☐	☒	The exact sample size (*n*) for each experimental group/condition, given as a discrete number and unit of measurement
☐	☒	A statement on whether measurements were taken from distinct samples or whether the same sample was measured repeatedly
☐	☒	The statistical test(s) used AND whether they are one- or two-sided<br>*Only common tests should be described solely by name; describe more complex techniques in the Methods section.*
☐	☒	A description of all covariates tested
☐	☒	A description of any assumptions or corrections, such as tests of normality and adjustment for multiple comparisons
☐	☒	A full description of the statistical parameters including central tendency (e.g. means) or other basic estimates (e.g. regression coefficient) AND variation (e.g. standard deviation) or associated estimates of uncertainty (e.g. confidence intervals)
☐	☒	For null hypothesis testing, the test statistic (e.g. *F*, *t*, *r*) with confidence intervals, effect sizes, degrees of freedom and *P* value noted<br>*Give P values as exact values whenever suitable.*
☒	☐	For Bayesian analysis, information on the choice of priors and Markov chain Monte Carlo settings
☒	☐	For hierarchical and complex designs, identification of the appropriate level for tests and full reporting of outcomes
☒	☐	Estimates of effect sizes (e.g. Cohen's *d*, Pearson's *r*), indicating how they were calculated

Our web collection on statistics for biologists contains articles on many of the points above.

Software and code

Policy information about availability of computer code

Data collection	R version 3.6.1 software was used for the analysis.
Data analysis	R version 3.6.1 software was used for the analysis.

For manuscripts utilizing custom algorithms or software that are central to the research but not yet described in published literature, software must be made available to editors and reviewers. We strongly encourage code deposition in a community repository (e.g. GitHub). See the Nature Research guidelines for submitting code & software for further information.

Data

Policy information about availability of data

All manuscripts must include a data availability statement. This statement should provide the following information, where applicable:

- Accession codes, unique identifiers, or web links for publicly available datasets
- A list of figures that have associated raw data
- A description of any restrictions on data availability

The datasets generated during and/or analyzed during the current study are available from the corresponding author on reasonable request.

Field-specific reporting

Please select the one below that is the best fit for your research. If you are not sure, read the appropriate sections before making your selection.

☒ Life sciences ☐ Behavioural & social sciences ☐ Ecological, evolutionary & environmental sciences

For a reference copy of the document with all sections, see nature.com/documents/nr-reporting-summary-flat.pdf

Life sciences study design

All studies must disclose on these points even when the disclosure is negative.

Sample size	This was a retrospective study with no sample size calculation. We initially identified 2645 consecutive patients with biopsy-proven breast cancer and initial image-proven brain metastases who were diagnosed at our institution between January 1 2002 and December 30 2015. We included patients with previously treated localized BC who are relapsing with 1-3 BMs without extracranial disease.
Data exclusions	These criteria were pre-specified. Patients with >3 BMs, or concurrent extracranial disease (n= 446), and duplicate or missing treatment data (n= 17) were excluded (Supplemental Figure.1).
Replication	To verify the reproducibility of our data, we compared the subtype distribution in our study (40% HER2+ and 28% triple negative (TN)) and showed that it was compatible with a study published previously by our group from a larger cohort of 873 patients with brain metastases showing 39.4% HER2+ and 34.1% TN subtypes (Gao et al Int J Cancer 2020)
Randomization	This was a retrospective study, so randomization is not applicable
Blinding	This was a retrospective study, so blinding is not applicable

Reporting for specific materials, systems and methods

We require information from authors about some types of materials, experimental systems and methods used in many studies. Here, indicate whether each material, system or method listed is relevant to your study. If you are not sure if a list item applies to your research, read the appropriate section before selecting a response.

Materials & experimental systems

n/a	Involved in the study	
☒	☐	Antibodies
☒	☐	Eukaryotic cell lines
☒	☐	Palaeontology and archaeology
☒	☐	Animals and other organisms
☒	☐	Human research participants
☐	☒	Clinical data
☒	☐	Dual use research of concern

Methods

n/a	Involved in the study	
☒	☐	ChIP-seq
☒	☐	Flow cytometry
☒	☐	MRI-based neuroimaging

Clinical data

Policy information about clinical studies

All manuscripts should comply with the ICMJE guidelines for publication of clinical research and a completed CONSORT checklist must be included with all submissions.

Clinical trial registration	not a clinical trial so not applicable
Study protocol	not a clinical trial so not applicable
Data collection	We identified 2645 consecutive patients with biopsy-proven breast cancer and initial image-proven brain metastases who were diagnosed at our institution between January 1 2002 and December 30 2015.
Outcomes	Overall survival (OS) was the primary outcome.<br>Progression free survival (PFS) and extracranial PFS were the secondary outcomes.
